# Improving childhood malaria treatment and referral practices by training patent medicine vendors in rural south-east Nigeria

**DOI:** 10.1186/1475-2875-8-260

**Published:** 2009-11-20

**Authors:** Theodora A Okeke, Benjamin SC Uzochukwu

**Affiliations:** 1 Department of Community Medicine, College of Medicine, University of Nigeria, Enugu Campus, Enugu, P.O. Box 3295, Nigeria

## Abstract

**Background:**

Malaria remains a major cause of morbidity and mortality among children under five years of age in Nigeria. Most of the early treatments for fever and malaria occur through self-medication with anti-malarials bought over-the-counter (OTC) from untrained drug vendors. Self-medication through drug vendors can be ineffective, with increased risks of drug toxicity and development of drug resistance. Global malaria control initiatives highlights the potential role of drug vendors to improve access to early effective malaria treatment, which underscores the need for interventions to improve treatment obtained from these outlets. This study aimed to determine the feasibility and impact of training rural drug vendors on community-based malaria treatment and advice with referral of severe cases to a health facility.

**Methods:**

A drug vendor-training programme was carried out between 2003 and 2005 in Ugwuogo-Nike, a rural community in south-east Nigeria. A total of 16 drug vendors were trained and monitored for eight months. The programme was evaluated to measure changes in drug vendor practice and knowledge using exit interviews. In addition, home visits were conducted to measure compliance with referral.

**Results:**

The intervention achieved major improvements in drug selling and referral practices and knowledge. Exit interviews confirmed significant increases in appropriate anti-malarial drug dispensing, correct history questions asked and advice given. Improvements in malaria knowledge was established and 80% compliance with referred cases was observed during the study period,

**Conclusion:**

The remarkable change in knowledge and practices observed indicates that training of drug vendors, as a means of communication in the community, is feasible and strongly supports their inclusion in control strategies aimed at improving prompt effective treatment of malaria with referral of severe cases.

## Background

More than a million people die of malaria each year and most of them occur in Africa [1]. In Nigeria, malaria is one of the leading causes of morbidity and mortality and a major public health problem with a prevalence rate of infection of 919/100,000. It accounts for 40% of disease burden (clinical out/inpatient cases) reported at the public health facilities, for 30% of all childhood deaths, and is associated with 11% of maternal deaths. The loss to the economy, as a direct result of malaria infections, has been estimated as Naira 132 billion (£530 million) [2]. Malaria is both a cause and a consequence of poverty in Nigeria.

In order to treat the disease, people seek treatment from a broad spectrum of public and private healthcare providers [3,4]. Around 60% of all malaria episodes in sub-Saharan Africa (SSA) are initially treated by private providers, mainly through the purchase of drugs from shops and drug peddlers [3-5]. Reasons for preferring drug shops include geographical accessibility, shorter waiting times, more reliable drug stocks, longer opening hours, friendly staff, greater confidentiality, lower cost and because they do not charge a separate fee for advice [6,7].

One of the problems associated with treatment by these patent medicine dealers is that there is poor knowledge and poor dispensing behaviour in relation to childhood malaria episodes [8]. These treatments are often incorrect [9-12] and the ineffectiveness of these early practices represents a major problem, since majority of malaria mortality occur within the first 48 hours of illness [13]. These practices may also contribute to the spread of anti-malarial drug resistance [14].

In Nigeria, patent medicine vendors (PMV's) are usually the first choice in health care and are a recognized primary source of orthodox drugs for both rural and urban populations, especially the poor [15-17]. In addition to selling drugs, they are also a major source of advice about illness and drug therapy [18].

The patent medicine vendor can be defined as a person without formal pharmacy training, who sells orthodox pharmaceutical products on a retail basis for profit [19]. The primary function of the PMV is a business and like any other successful business person, the PMV tries to respond to customer demand, hence the bulk of customer-PMV interactions simply involve the selling of medications requested by the customer [20]. Over-the-counter (OTC) drugs are the only drugs authorized to be sold by the vendors, but they generally sell all types of drugs as determined by their financial capability [21]. These range from paracetamol and chloroquine in large tins to antibiotic, psychotropic drugs, narcotics, toxoids, and antihypertensives, that are outside the scope of their license [22]. In addition, the PMV obtains their drug supplies through both formal and informal channels including large retail and wholesale pharmacies in major cities, direct from pharmaceutical companies, and through visiting company representatives [22]. There are also reports that these drugs may be ineffective, counterfeit or expired [21].

In addition, most of the anti-malarial drugs purchased from PMVs in a study in Nigeria were for children [23]. Since private drug retailers are often the primary source of treatment for malaria in the home, this underscores the need for improved practices within these outlets, taking into consideration the problems associated with using them.

The results of the formative stage (pre-intervention phase) of this study [8] showed that there was poor knowledge and poor dispensing practices in relation to childhood malaria among these drug vendors in the study area, although referral of severe malaria was common there were those that will not refer to a health facility. History was rarely taken before dispensing drugs and verbal advice was never given to care takers.

Based on these results an intervention programme was developed to determine the feasibility of training drug vendors to provide appropriate treatment for childhood malaria, to take on an advisory role and to refer severe cases of malaria promptly, with a view to reducing childhood morbidity and mortality and achieving the health related Millennium Development Goals 4.

Thus this report is part of a larger study designed to improve home management of childhood malaria and referral of severe malaria at the household and community levels.

## Methods

### Study area

The study area has previously been described [8]. Malaria is holoendemic with a high transmission rate all the year round and an average incidence rate of 20%. The main malaria vector is *Anopheles gambiae *and *Plasmodium falciparum *is responsible for more than 90% of all malaria infection [24]

### The intervention programme

The intervention programme commenced on the 30^th ^December 2003 for a period of 18 months, until June 2005. The total number of drug vendors had increased from 13 at time of baseline study[8] to 16 by intervention period and all belonged to the PMV association.

The main components of the programme were training of drug vendors, community information activities, monitoring and evaluation of the programme. In recognition of the prominent role which traditional, administrative and political leaders play in ensuring community participation and successful implementation of an intervention programme, a series of sensitization meetings were held in the community. Having obtained the support of the community leaders, meetings were then held with the members of drug vendors association to gain their cooperation. Enough trust was built with the drug vendors during the formative phase. In addition, the results of the formative phase were fed back to them and they showed great interest in the intervention.

A core group known as Project-Community Communication Group (PCCG) was formed. The composition of the PCCG included women leaders, opinion leaders, representatives of patent medicine vendors and project members. The main aim was to promote community participation and for the group to help in the design, implementation and monitoring of the project and also to act as a link between the research team and the community.

**Figure 1:**
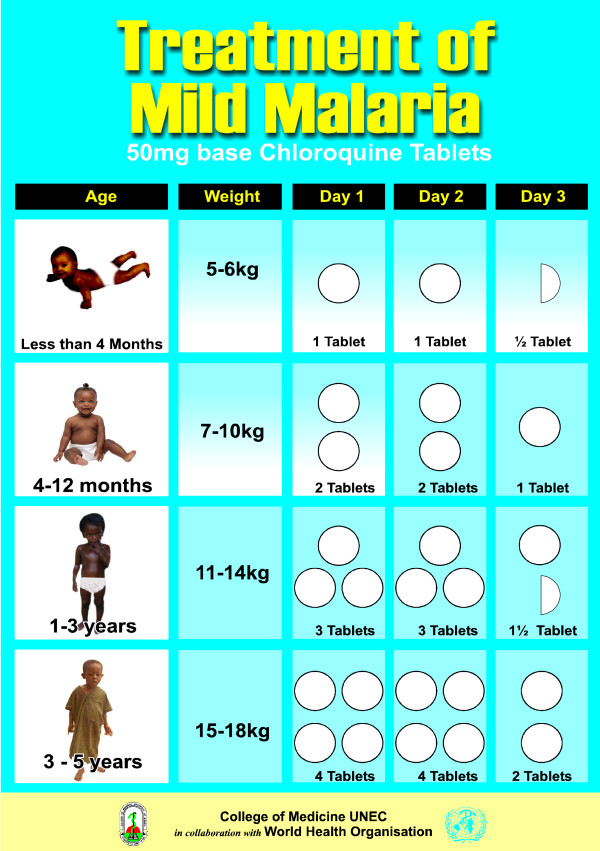
**Pictogram of chloroquine dosing schedule for children of different ages**. Age groups are identified by pictures of children at different developmental stages, which is easier to recognize than exact ages or weights. The number of tablets is also represented pictorially.

Some members of the research team, representatives of drug vendors and a graphic artist worked together to design the job aids which included, age-specific dose guides for treatment of malaria with chloroquine (Figure 1) and paracetamol. Age groups are identified by pictures of children at different developmental stages, which are easier to recognize than exact ages or weights. The number of tablets is also represented pictorially.

Age-specific rubber stamps depicting correct doses of chloroquine in children of different ages with different colours of handles for the different age groups for easy recognition were produced. Posters were also produced depicting a child with mild and severe malaria with a list of the clinical symptoms. This was to aid drug vendors in diagnosis of severe malaria requiring referral. It was also to serve as client awareness poster to help caretakers differentiate between mild and severe malaria

The community information activities aimed to create awareness of the programme, give information on recommended anti-malarial drug policy, importance of appropriate early treatment and symptoms of severe disease requiring referral to a health facility. This was done through community meetings, distribution of posters, which were placed in strategic places in the community, and drama groups, which performed regularly. The results of this will be published elsewhere.

### Training of drug vendors

Drug vendors were trained in a series of two workshops each lasting three days. To ensure full participation, all the training activities took place on days that were not market days, when they were considered very busy. The participatory approach was adopted for the training, which emphasized culturally appropriate methods, such as role play.

Training focused on improving their age-specific dispensing practices for chloroquine, which was the recommended anti-malarial at time of study, asking relevant history questions before giving treatment instructions as well as effective communication skills to enable them give accurate verbal advice to caretakers. They were trained to recognize symptoms requiring referral and were taught to record all referrals on a notebook by writing the child's name, the date and reasons for the referral. Referral was facilitated by giving the caretakers referral note stamped with a referral stamp and also by informing nearby hospitals to recognize the referral stamp as that of a child requiring urgent attention.

The caretakers were to choose the hospitals of their choice, since baseline results showed the local health centre was not being utilized for reasons that included the lack of a doctor. To measure compliance with referral, some community members chosen by the PCCG, were detailed to follow up the referred patients at home to confirm compliance.

In addition, sessions on reading the doctor's prescription was included based on drug vendors request and the fact that patients will eventually end in their shops to purchase drugs. At the end of the workshop, they were given job aids such as age-specific dose guides for malaria treatment to be hung up on the wall of their shops, to serve as reminders before dispensing drugs. They were taught to stamp the dispensing envelopes with the right dose for the age of sick child, to serve as a visual reminder of the verbal advice given to caretakers.

At the end of the training, certificates of participation were distributed to all participants. Drug vendors appreciated the certificates as this will elevate their status within the community as well as boost sales of drugs. They were neither charged nor remunerated for their participation.

### Monitoring and evaluation

This was carried out by some members of the research team and trained research assistants. Monitoring and face-to-face supportive supervision for drug vendors, designed to reinforce skills was carried out for a period of eight months. Unannounced face-to-face observations were conducted to monitor health care providers performance after training. Later supportive feedback was provided for problems they were not aware of and important issues raised were solved to help them integrate the new knowledge and skills into their daily practices.

The programme was evaluated by conducting three rounds of exit interviews on all trained PMVs. Immediately after leaving the PMVs' outlet, the caretaker was interviewed at a location away from the shop, using a pre-structured form, which was developed to record all the behaviours the PMVs were expected to perform at the end of training. This is with regards to history questions asked, treatment given, whether reference was made to the dose guides provided, if stamped envelopes were given and type of verbal advice given if any.

In-depth interviews (IDI) were conducted with the PMVs using IDI guide to explore their knowledge, beliefs, and stated treatment practices for mild and severe malaria, as well as the referral practices for severe malaria. The interview was conducted in the morning hours on non-market days (when the patient flow to the drug sellers' stores was minimal) by two community health nurses from the Health Visiting Unit of the Department of Community Medicine, who also conducted the pre-intervention phase interviews. Each interview lasted between 60 and 75 minutes. The interview was tape-recorded and notes taken.

In addition, a review of the PMVs' referral records was conducted. The review noted the total number of cases referred during the study period by drug vendors and the main reasons for referral. The places the patients were referred to was also noted. To measure compliance with referral, some community members chosen by the PCCG, were detailed to follow up the referred patients at home to confirm compliance and this information was collected from the PCCG members.

### Data analysis

Data entry and statistical analysis was conducted using EPI-INFO version 6.04 and SPSS statistical packages version 15. Chi-square test was used to test for any significant differences between proportions before and after intervention.

For the qualitative data, the transcriptions was organized under thematic headings and later developed into an ethnographic summary with illustrative quotes. The trajectories of their response to questions were captured with a trajectory tree and then the different ranges of opinions, perceptions and stated practices were noted.

### Ethical considerations

Ethical approval was sought and obtained from the Ethical Committee of The University of Nigeria Teaching Hospital, Enugu (UNTH). The research objectives and methods were explained to individual respondents and verbal informed consent to tape-recording the interviews was obtained from the study participants before the interview commenced.

Confidentiality of all information obtained from participants was maintained by not allowing information on the respondents' identities to be accessible to non-members of the research team.

## Results

A total of 16 patent medicine vendors (PMVs), registered with the association were trained. They comprised eleven male and five female practitioners and the age range was from 26 to 42 years. The majority (14/16, 87.5%) had attained primary education while only (2/16, 12.5%) had secondary education. Only one, who is a trained nurse, had received any formal training in drug use. Generally, they were willing to participate in the programme and all 16 of them were trained giving 100% participation rate.

They acknowledged the importance of profit as a motive for their drug dispensing habit, preferring a sale of single tablets (incomplete course) to no sale at all. However, they were advised to sell only full courses of anti-malarials to caretakers and the sale of single tablets was discouraged. They were eager to receive further training and to broaden the scope of their therapeutic activities.

The result of the exit interviews is shown in Table 1. There was a steady increase in correct practices over time and this was found to be statistically significant, p < 0.05. As the months went by, more drug vendors asked correct history questions, made reference to dose guides before dispensing anti-malarials, gave correct treatment of chloroquine accompanied by accurate verbal advice and stamped dispensing envelopes with correct rubber stamps for age of the sick child.

**Table 1:** Exit interview for drug vendors

	1^st ^month (n = 190)	4^th ^month (n = 100)	8^th ^month (n = 188)	X^2 ^for trends P value
Correct history questions asked	83 (43.7%)	75 (75%)	173 (92%)	101.3 p < 0.001*

Reference to dose guides	80 (42.1%)	69 (69%)	171 (90.9%)	100.7 P < 0.001*

Correct treatment given	80 (42.1%)	69 (69%)	171 (90.9%)	100.7 p < 0.001*

**Correct verbal advice	71 (37.3%)	63 (63%)	167 (88.8%)	106.7, p < 0.001*

Stamped envelope given	83 (43.7%)	63 (63%)	174 (92.6%)	102.6, p < 0.001*

* = significant.

** = correct advice was regarded as (telling clients how to take their drugs, side effects of the drugs and what to do if symptoms persists)

### Causes and mode of transmission of malaria

During the pre-intervention phase [8], some of the PMVs had more than one perception about the cause of malaria. These include mosquitoes (10/13, 76.9%), sun (6/13, 46.2%) and drinking dirty water (4/13, 30.8%). Other causes mentioned by less than four vendors, included eating fried/oily food, cold and body contact. When asked to state how the sun causes malaria, one of them explained " *the sun can cause malaria when it shines directly on the child*". However, after the intervention, (15/16, 93.8%) of drug vendors knew mosquitoes as a cause of malaria, (1/16, 6.3%), still attributed malaria to eating of oily/fried food. In the pre-intervention, the mode of transmission was also not quite clear. However, in the post-intervention phase, the majority had acquired correct knowledge about mode of transmission. According to one vendor" *when mosquito bites a person carrying the parasite already, it carries the parasite and transfers it to a healthy person when it bites that person. In that way it transfers the parasite from a sufferer to a healthy person and malaria starts"*.

### Symptoms and signs of malaria

During the pre-intervention phase, when asked to discuss the symptoms of malaria, drug vendors correctly identified them to be fever (9/13, 69.2%), headache (8/13, 61.5%), weakness (7/13, 53.8%), loss of appetite (7/13, 53.8%) and restlessness (6/13, 46.2%) for mild malaria. Other less commonly noted symptoms included deep yellowish discoloration of urine, chills, vomiting, and stomach upset, dreaming, and excessive sweating. However, following training, there was an increase in recognition of malaria as (14/16, 87.5%) of vendors identified fever as a major symptom of malaria followed by loss of appetite (68.8%), chills (62.5%), headache (62.5%), weakness (56.3%), and vomiting (50%). They were less knowledgeable about symptoms of severe illness before intervention. Persistent fever and convulsions, which were recognized by 38.5% and 30.8% of vendors, respectively, were the popular answers. Also mentioned were yellowish discoloration of the skin (23.1%) and unconsciousness (15.4%). But the intervention had a positive impact on the recognition of signs of severe malaria. In the post-intervention, most drug vendors could now list the cardinal signs, such as convulsions (87.5%), persistent fever (68.8%), unconsciousness (62.5%), dryness of skin (62.5%), inability to stand/sit (56.3%), yellowish discoloration of the skin (50%), fast breathing (43.7%) and coke-colored urine (43.8%). The relationship between malaria and convulsion, which was previously vague, was also better understood as shown in this quote "*malaria gives very high fever and convulsion occurs from very high fever, again when malaria enters the brain of the child, it causes convulsion"*

### Treatment practices

During formative research, when asked about how treatment decisions are made (8/13, 61.5%) responded they dispensed what customers demand. Only (2/13, 15.4%) did take some form of history, by asking about age of the child only, before giving drugs and none gave any verbal advice to caretakers. Furthermore, a majority (11/13, 84%) used chloroquine as their first-line drug, in addition to analgesics in the form of paracetamol, as an adjunct to specific anti-malarial drugs for the treatment of mild malaria. In general the knowledge with regards to drug dosages and duration of treatment was grossly inadequate and none dispensed anti-malarial drugs correctly.

However, the intervention resulted in an increase in knowledge and correct practices as shown by results of exit interviews in Table 1 and the qualitative data. The number that will take a history rose from 43.7% in the first month to 75% by the fourth month after training and to 92% after 8 months. A female drug vendor in an IDI commented that "*you do not commence treatment immediately, you first take history, if he has not received any treatment you then give them some drugs and tell them how to take it." *The majority also refered to their age-specific dose guides before administering any treatment, as one of them noted "*when the mother brings the child, I will welcome the mother, sit her down, ask the mother when the sickness started, what type of treatment she has given. Then after this I will bring the chart so that I will give the correct dose according to the age of the child*. Yet another remarked "*I look at the drug guides you gave us to treat these children"*. Commenting on the efficacy of treatment one of them observed that *"if they bring the child on time this treatment works fast and the child recovers immediately after 3 days"*.

The percentage of anti-malarial drug sales where an adequate amount of chloroquine was sold rose from 42.1% one month after training to 69% after four months and to 90.9% after 8 months. It was noted that the same drug vendors who made reference to their dose guides also dispensed chloroquine correctly. Generally there was a steady increase in correct practices over the months with regards to history questions asked, reference to dose guides, treatment given, verbal advice and stamping of dispensing envelopes. All these observed differences were statistically significant (p < 0.05) as shown in table 1.

### Referral practices

Prior to the intervention, most drug vendors indicated that they also made use of chloroquine and paracetamol as the major drugs for the treatment of severe malaria. None did refer a child with severe malaria to a health facility, unless he starts convulsing. There was a gap in knowledge of severe symptoms requiring referral. After the intervention, most had learnt that severe cases should be referred immediately. As one vendor remarked *"if it is severe malaria it is above my scope, I will tell them to go to the hospital. You cannot treat this type because it is beyond my scope"*. Yet another explained *"when we use the system we learnt from the training and treat mild malaria, if it does not respond, we refer them to the hospital on time"*.

A total of 132 cases were referred during the study period by drug vendors out of which 115 (80%) complied. The main reasons for referral were high fever, weakness and vomiting. A majority 50 (47.6%) of the referred patients went to Parklane General hospital, while 37 (35.2%) of them went to the University of Nigeria Teaching Hospital, both situated in the urban area about 20 kilometers away. Nine (8.6%) used private clinics and the community health center. All severe cases were referred.

## Discussion

The training interventions were aimed at building the knowledge and skills that will enable PMVs to provide better treatment for malaria and refer severe malaria cases. This study shows that the training improved their knowledge and correct practices. These results are rather encouraging, considering the difficulty involved in changing private sector practices. As noted by a WHO study group, achieving rational prescribing in the private sector is "notoriously difficult" due to influences from patient demand, drug advertising and profit margins [25].

A majority of them were able to provide anti-malarials in the right dose and for the right duration. No doubt this is likely to improve the effectiveness of anti-malarial treatment, and hence reduce the development of drug resistant and eventual cost of treatment. The result is similar to what was obtained in Kenya [26], where peer education supplemented with job aids was able to produce improvements in the selling of appropriate drugs in appropriate amounts.

The intervention also showed that drug vendors were able to give correct verbal advice to their patients in such a way that the customers understand and can follow the advice. However, as has been noted in Nigeria [20], there may be concern that profit motive might muffle the interest in medication counseling as the vendors may not want to waste their time giving advice to the clients when there is rush in their shops.

The referral of severe cases of malaria to a health facility also improved. This is particularly encouraging as increased trend in morbidity from severe malaria has been noted in Nigeria [27] and early referral from PMVs is likely to improve the outcome of severe malaria.

The training programme focused on chloroquine because at the time of the study it was the recommended first-line treatment and the only anti-malarial drug available in the majority of shops in the study area. The new policy, which recommended a change to artemisinin-based combination therapy (ACT), was officially inaugurated in May 2005 [28], by which time the intervention programme had ended. The policy change became necessary as the therapeutic efficacy of chloroquine had deteriorated. However, the choice of drug in this study was not as pertinent as establishing the fact that community education on drug use and referral of severe cases, can be effectively carried out by patent medicine vendors.

The improvement in the knowledge of the vendors suggests that they are very receptive and that their training is feasible. They are, therefore, likely to be useful in health interventions when given the appropriate training.

The interpretation of results of this study is limited by the fact that it lacks randomization. Nevertheless the data shows a highly probable relationship between the introduction of a specific training programme and changes in PMV's behavioural practices, which are strongly related to the content of the intervention. In addition there were no other local sources of information on anti-malarials during the study period, other than biased information from pharmaceutical companies.

Generally the advantages of training to drug vendors would be a possible increase in prestige and OTC sales, especially if certificates were issued at the end of the training as was the case in our study. The advantages to rural communities, would be that they would receive more reliable advice when they purchase OTC drugs. If carried out effectively such a programme could reduce morbidity and mortality in these communities, by initiating prompt appropriate treatment of malaria with early referral of severe cases to a health facility. The potential role of private retailers as partners for malaria control has long been recognized [29-33] and success has been achieved in training them [20,26] and it was shown that PMVs receiving job aids had significantly better malaria knowledge and prescribing practices than those that did not [34]. However, key challenges to lasting success in Nigeria, are issues such as: high vendor turn-over rates in shops, sustainability of the change, need for continuous re-training and patient compliance with recommended treatment regimens

## Conclusion

The primary goal of malaria control is to reduce morbidity and mortality through prompt diagnosis and adequate treatment. The focus of delivery of such service has been through the formal health facilities. However, such settings, where treatment is provided by trained personnel, represents only a small part of the providers of care for malaria patients

The retail sector seems to have the greatest potential in improving access to anti-malarials. This is because this sector is generally more accessible geographically and treatment from this source is cheap and involves less time.

The findings of this study strongly support the inclusion of patent medicine vendors in malaria control strategies in form of public private partnership and are in line with the philosophy of primary health care, which is noted for advocating incorporation of local human resources into healthcare efforts. This was demonstrated in Tanzania [35] and in Nigeria with other health interventions [36]. Drug vendors should, therefore, be made partners in the health care network, since they are likely to continue as a major source of anti-malarial drugs for most rural communities in the foreseeable future.

With the newly introduced high cost ACT, a policy option to improve diagnosis and prescribing pattern in private sectors will be to train PMVs in the appropriate use of ACT, using pictorial aids and dose guide as in this study. Although there is no guarantee that the PMVs will perform as well as with chloroquine, it is believed that If well taken by the PMVs, it is likely to decrease inappropriate drug prescribing, use, costs and resistance to ACT. It may also enhance the delivery of ACT for malaria treatment.

## Competing interests

The authors declare that they have no competing interests.

## Authors' contributions

OT and UB conceived and designed the study, collected the data, analysed and wrote up the manuscript.
